# Effect of Hypoxia-Ischemia on the Expression of Iron-Related Proteins in Neonatal Rat Brains

**DOI:** 10.1155/2023/4226139

**Published:** 2023-04-20

**Authors:** Qing Lin, Ding-Wang Hu, Xin-Hui Hao, Geng Zhang, Ling Lin

**Affiliations:** ^1^Laboratory of Clinical Applied Anatomy, Department of Human Anatomy, School of Basic Medical Sciences, Fujian Medical University, Fuzhou 350122, China; ^2^Key Laboratory of Brain Aging and Neurodegenerative Diseases of Fujian Province, Fuzhou 350122, China; ^3^Public Technology Service Center, Fujian Medical University, Fuzhou 350122, China

## Abstract

Hypoxic-ischemic white matter injury (WMI) pathogenesis in preterm infants is not well established, and iron-related proteins in the brain may play an important role in imbalanced iron metabolism. We aimed to investigate the iron-related protein changes in neonatal rats after hypoxia-ischemia (HI), clarify the role of iron-related proteins in hypoxic-ischemic WMI, and potentially provide a new target for the clinical treatment of hypoxic-ischemic WMI in preterm infants. We adopted a WMI animal model of bilateral common carotid artery electrocoagulation combined with hypoxia in neonatal 3-day-old Sprague-Dawley rats. We observed basic myelin protein (MBP) and iron-related protein expression in the brain (ferritin, transferrin receptor [TfR], and membrane iron transporter 1 [FPN1]) via Western blot and double immunofluorescence staining. The expression of MBP in the WMI group was significantly downregulated on postoperative days (PODs) 14, 28, and 56. Ferritin levels were significantly increased on PODs 3, 7, 14, and 28 and were most significant on POD 28, returning to the sham group level on POD 56. FPN1 levels were significantly increased on PODs 7, 28, and 56 and were still higher than those in the sham group on POD 56. TfR expression was significantly upregulated on PODs 1, 7, and 28 and returned to the sham group level on POD 56. Immunofluorescence staining showed that ferritin, TfR, and FPN1 were expressed in neurons, blood vessels, and oligodendrocytes in the cortex and corpus callosum on POD 28. Compared with the sham group, the immune-positive markers of three proteins in the WMI group were significantly increased. The expression of iron-related proteins in the brain (ferritin, FPN1, and TfR) showed spatiotemporal dynamic changes and may play an important role in hypoxic-ischemic WMI.

## 1. Introduction

Preterm newborns have high incidence and morbidity rates. White matter injury (WMI) is a common form of brain damage in premature birth survivors [1, 2]; the typical pathological change is dysmyelination [3]. To date, the pathogenesis of WMI has not been completely elucidated.

WMI caused by hypoxia-ischemia (HI) may be related to oxidative stress [4], elevated intracellular calcium concentration [5], and inflammation [6]. As an essential trace element in energy metabolism and DNA and protein synthesis, iron plays a key role in neuronal damage caused by oxidative stress during HI [7, 8]. The changes in circulating iron levels may cause iron accumulation in the nervous system [9], and excessive iron accumulation causes neurodegenerative changes that contribute to damage to neurons, astrocytes, and cerebrovascular endothelial cells [10]. Clinical studies have shown that obvious bleeding points appear in the periventricular white matter after WMI in newborns [11], and abnormal accumulation of iron content appears in the basal ganglia [12] while non-protein bound iron levels in the cerebrospinal fluid and serum are significantly increased [13]. Our previous study results also indicate that the iron content in the brains of neonatal rats increased significantly after HI [14]. The above results suggest that the iron metabolism changes may be involved in the occurrence and development of neonatal WMI, although the specific process and mechanism of abnormal iron metabolism are not completely understood.

Iron homeostasis is maintained by the balance of iron uptake, usage, and storage, which is closely related to iron metabolism-related proteins [15, 16]. Iron-related proteins mainly include ferritin, transferrin receptor (TfR), and membrane iron transporter 1 (FPN1). Ferritin, the main iron storage protein, is a high molecular weight water-soluble protein that can store 2000–4500 iron ions [17]. The TfR family includes TfR1 and TfR2, which are composed of 760 and 801 amino acids, respectively. TfR1 and TfR2 are non-heme iron membrane surface input proteins that can combine with transferrin to form a complex for endocytosis in the cell body and facilitate the absorption and release of iron between the cell membrane and the nucleus [18]. FPN1 is an important transmembrane iron export protein. It is the only known cellular iron exporter in vertebrates. Under the action of hepcidin, the amount of dietary iron, circulating iron, and stored iron released into the plasma could be controlled by changing its distribution on the cell membrane, thus maintaining iron homeostasis [19].

There are an increasing number of studies on iron metabolism-related proteins in brain diseases. In a middle cerebral artery occlusion stroke model in rats, ferritin levels increased significantly, and FPN1 levels decreased significantly in the cerebral cortex, hippocampus, and striatum on the ischemic side. Moreover, the TfR levels increased significantly in the cerebral cortex and hippocampus [7]. Iron accumulation was also present in the hippocampus of chronic intermittent hypoxic mice, accompanied by high levels of ferritin and TfR and low levels of FPN1 [20]. Giesinger et al. also observed increased expression of ferritin, FPN1, and TfR in astrocytes and microglia after hypoxia [21]. These studies showed that iron-related proteins play an important role in the process of iron metabolism. Therefore, when HI occurs, how do the iron-related proteins change in the brain? At present, no comprehensive study has been conducted on this topic. Discussion of this problem will help clarify the mechanism of brain iron imbalance during hypoxia and ischemia and provide a theoretical basis for the development of therapeutic drugs targeting these proteins.

The aim of our study was to use a hypoxic-ischemic WMI animal model with 3-day-old Sprague-Dawley (SD) rats to explore the temporal and spatial dynamic changes of iron metabolism-related protein expression (ferritin, TfR, and FPN1) in the brains of hypoxic-ischemic WMI rats and clarify the possible role of iron metabolism-related proteins in hypoxic-ischemic WMI.

## 2. Materials and Methods

### 2.1. Animals

We used specific pathogen-free SD rats (male or female) that were 3 days postnatal (P3), which were randomly divided into WMI and sham groups. The rats were housed in a temperature-controlled (22–25°C) barrier environment with free access to food and water. The present study complied with the rules for the use of experimental animals and was approved by the Animal Ethics Committee of Fujian Medical University (Animal License Number: SYXK [Min] 2016-0006).

### 2.2. WMI Model

As described in the study by Wang et al. [22], the experimental WMI model in newborn SD rats was induced using the hypoxic-ischemic method or sham operation. Briefly, SD rats at P3 were anesthetized with isoflurane and were fixed on the operating table in a supine position, and the neck skin was disinfected with 75% alcohol. Bilateral common carotid arteries were separated in both groups and cut off using electric coagulation forceps in the WMI group but without coagulation in the sham group. After surgery, the rats were reunited with their mothers for breastfeeding. Two hours later, the WMI group was placed in a self-made hypoxic chamber (submerged in a 37°C water bath to maintain normothermia) through which a humidified gas mixture (8% oxygen balanced with nitrogen) flowed for 30 min, while the sham group was placed in a different container for normoxic treatment. Finally, the rats were returned to their mothers for further feeding.

### 2.3. Western Blot

The rats were anesthetized with isoflurane and perfused with normal saline; the collected brain samples were taken to extract proteins. The samples were homogenized and centrifuged for 20 min (4°C, 12,000 rpm). The supernatant was collected to analyze the protein concentration. Proteins were separated using sodium dodecyl sulfate-polyacrylamide electrophoresis and transferred to polyvinylidene fluoride (PVDF) membranes (Millipore, Billerica, MA, USA). PVDF membranes were blocked with 5% defatted milk for 2 hours. Then, the membranes were incubated overnight at 4°C with the primary antibody dilution (Meilunbio, MB9881) containing rabbit anti-myelin basic protein (1 : 1000, CST, 78896s), rabbit anti-ferritin (1 : 1000, Abcam, ab75973), mouse anti-transferrin receptor (1 : 1000, Invitrogen, 136800), rabbit anti-ferroportin (1 : 500, NOVUS, nbp1-21502), and rabbit anti-ACTB antibody (1 : 2000, BBI, D110001-0100). Then, PVDF membranes were incubated with secondary antibodies including goat anti-mouse IgG HRP (1 : 5000, BIOSS, bs-0296G-HRP) and goat anti-rabbit IgG HRP (1 : 5000, BIOSS, bs-0295G-HRP) for 2 hours. After washing the membrane as previously described, the bands were visualized and photographed using a gel imager (Bio-Rad, USA). The gray value of every band was analyzed using ImageJ software and presented as the relative optical density of *β*-actin. Error bars represented SD. Data were from four independent experiments.

### 2.4. Immunofluorescence Staining

The rats were anesthetized with isoflurane and perfused with normal saline followed by 4% paraformaldehyde. The brain samples were obtained and postfixed in 4% paraformaldehyde for 30 min followed by saturation in a 20% sucrose solution for 24 hours, then a 30% sucrose solution for 48 hours. The tissues were covered with an optimal cutting temperature compound and cut into 14 *μ*m thick coronal slices using a cryostat (Leica, Buffalo Grove). The sections were washed three times with phosphate buffer solution (PBS) and then blocked in QuickBlock™ blocking buffer for immunol staining (Beyotime, P0260) for 30 min. Sections were then incubated overnight at 4°C with primary antibodies diluted in PBST: rabbit anti-MBP (1 : 200, CST, 78896s), rabbit anti-ferroportin (1 : 200, NOVUS, nbp1-21502), mouse anti-transferrin receptor (1 : 200, Invitrogen, 136800), rabbit anti-ferritin (1 : 100, Abcam, ab75973), mouse anti-NeuN (1 : 200, Abcam, ab104224), rabbit anti-NeuN (1 : 200, Abcam, ab177487), mouse anti-CD31 (1 : 250, Millipore, mab1393I), rabbit anti-CD31 (1 : 250, Abcam, ab281583), mouse anti-Olig2 (1 : 200, Sigma, MABN50), and rabbit anti-Olig2 (1 : 200, CST, 65915T). Sections were washed in PBST and incubated with the fluorescent-labeled secondary antibodies including donkey anti-rabbit IgG (Alexa Fluor 555, 1 : 500, Abcam, ab150062) and goat anti-mouse IgG (Alexa Fluor 488, 1 : 500, Abcam, ab150117) for 1 hour at room temperature. Sections were subsequently washed in PBST and DAPI (CST, 4083). The results were visualized and photographed under a confocal laser-scanning microscope (Leica, TCS SP8, Germany).

### 2.5. Statistical Analysis

Data were expressed as means ± standard deviations. Statistical analyses were performed using SPSS 21.0 (IBM, Armonk, NY, USA). GraphPad Prism 8 software (GraphPad, Inc., La Jolla, CA, USA) was used to plot the expression curves of different proteins at different time points. Statistical differences were calculated using Student's *t* test with two tails and two-way ANOVA. *p* < 0.05 was considered statistically significant.

## 3. Results

### 3.1. After HI, MBP Expression Was Downregulated Confirming the Occurrence of WMI

**Figure 1:**
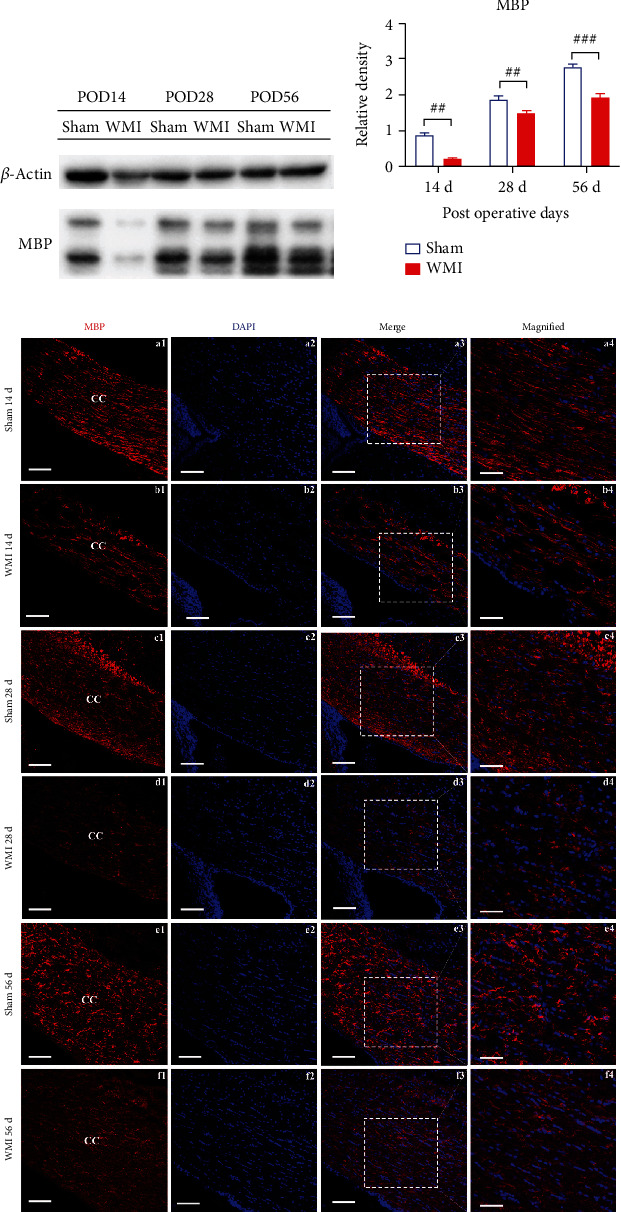
Effect of hypoxia-ischemia on the expression of MBP in rat brains. (a) Representative Western blot images of MBP bands and PODs (postoperative days). (b) Quantitative analysis of MBP protein expression (^##^*p* < 0.01 and ^###^*p* < 0.001 vs. sham group, *n* = 4). The results showed that the MBP level was clearly reduced in the WMI group compared with the sham group. (c) Double immunofluorescence staining of MBP (red) and DAPI (blue). Among them, a3, c3, and e3 are the high-magnification images in the white line square areas of b3, d3, and f3. CC: corpus callosum; a1–a3, b1–b3, c1–c3, d1–d3, e1–e3, f1–f3, bar = 50 *μ*m; a4, b4, c4, d4, e4, f4, bar = 25 *μ*m.

MBP is the main component of the myelin sheath and can be used as an important marker to determine myelin sheath injury. To observe the effect of HI on the myelin sheath, we used Western blot and immunofluorescence staining to observe the changes in MBP expression. The results showed that, compared with the sham group, MBP expression in the WMI group was significantly downregulated on PODs 14 (*p* < 0.01), 28 (*p* < 0.01), and 56 (*p* < 0.001) (Figures 1(a) and 1(b)), and the fluorescence intensity of MBP immunopositive substances in the corpus callosum was significantly decreased (Figure 1(c)). These results suggest that WMI occurred after HI in neonatal rats.

### 3.2. Expression of Iron-Related Proteins in the Brain Changed Dynamically after HI in Neonatal Rats

**Figure 2:**
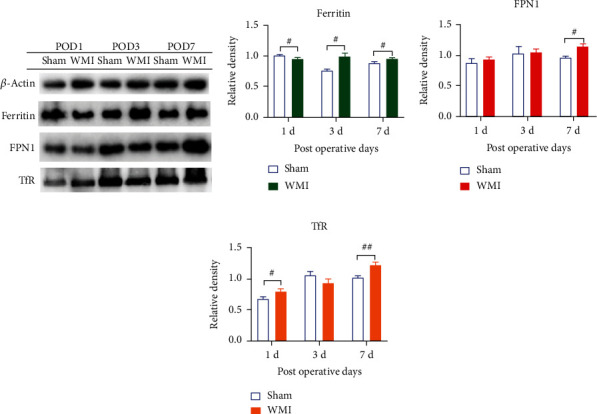
Effect of hypoxia-ischemia on the expression of ferritin, FPN1, and TfR in brain tissue on PODs 1, 3, and 7. (a) Bands of Western blots from brain tissue. (b–d) Relative protein expression of ferritin, FPN1, and TfR (^#^*p* < 0.05 and ^##^*p* < 0.01 vs. sham group, *n* = 4).

**Figure 3:**
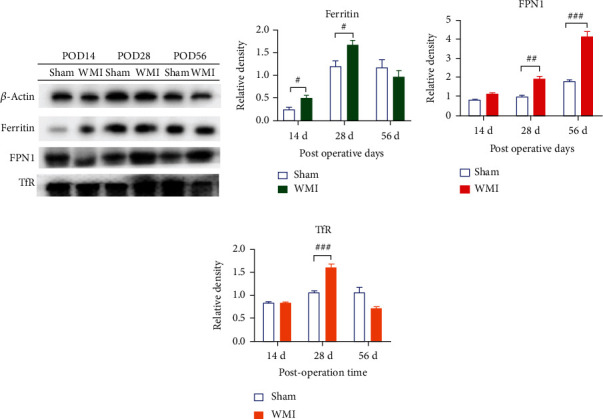
Effect of hypoxia-ischemia on the expression of ferritin, FPN1, and TfR in brain tissue on PODs 14, 28, and 56. (a) Bands of Western blots from brain tissue. (b–d) Relative protein expression of ferritin, FPN1, and TfR (^#^*p* < 0.05, ^##^*p* < 0.01, and ^###^*p* < 0.01 vs. sham group, *n* = 4).

Previous studies have found that iron metabolism in the neonatal rat brain was dynamically altered after HI. Therefore, we considered whether the iron-related proteins that maintain iron metabolism balance were altered during WMI. To confirm this, Western blot was used to detect the expression of iron-related proteins (ferritin, TfR, and FPN1). The results showed that, compared with the sham group, ferritin expression in the WMI group was decreased on POD 1 (*p* < 0.05) (Figure 2(b)) and increased on PODs 3, 7, 14, and 28 (*p* < 0.05) (Figures 2(b) and 3(b)). The expression of FPN1 in the WMI group was increased on PODs 7 (*p* < 0.05), 28 (*p* < 0.01), and 56 (*p* < 0.001) (Figures 2(c) and 3(c)). The expression of TfR in the WMI group was significantly upregulated on PODs 1 (*p* < 0.05), 7 (*p* < 0.01), and 28 (*p* < 0.001) as shown in Figures 2(d) and 3(d).

**Figure 4:**
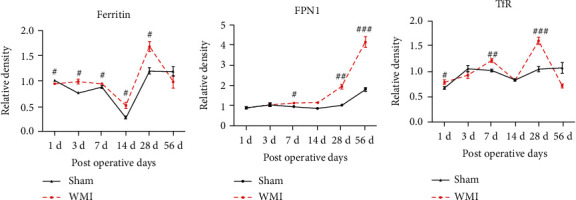
Changes in the expression of ferritin, FPN1, and TfR in brain tissue over time (^#^*p* < 0.05, ^##^*p* < 0.01, and ^###^*p* < 0.01 vs. sham group, *n* = 4).

Then, we analyzed the expression of iron-related proteins in both groups at different time points, and the results showed that the change trends were basically the same. The expression of ferritin decreased initially and then increased. The expression was the lowest on POD 14, peaked on POD 28, and returned to the level of the control group on POD 56, as shown in Figure 4(a). The expression of FPN1 increased with time during the 56 days after HI, as shown in Figure 4(b). The expression of TfR showed an upward trend from POD 1 to POD 7 and an initially upward trend followed by a downward trend from POD 14 to POD 56. As shown in Figure 4(c), the result was most obvious on POD 28 and returned to the level of the control group on POD 56.

### 3.3. Changes in the Localization and Expression of Iron-Related Proteins after HI in Neonatal Rat Brains

**Figure 5:**
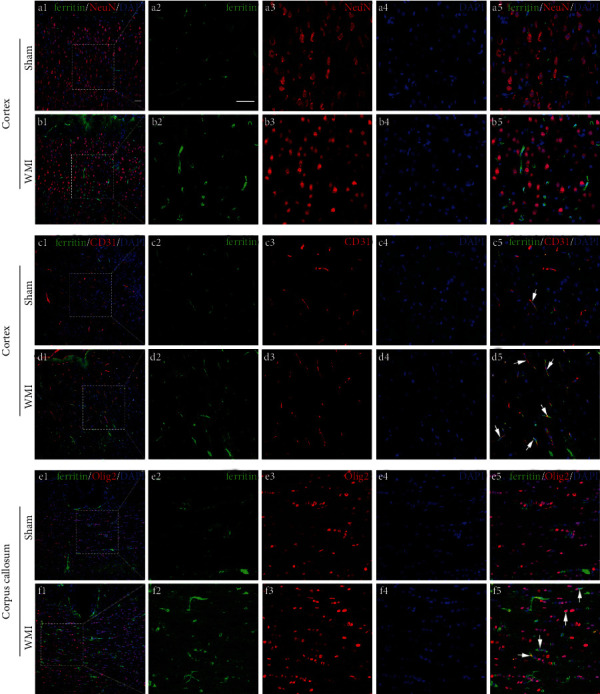
Representative images of immunofluorescence staining for ferritin (green), NeuN (neuronal marker; red)/CD31 (vascular marker; red)/Olig2 (oligodendrocyte marker; red), and DAPI (nuclear marker; blue) in rat brains on POD 28. Ferritin-positive cells were colocalized with CD31- or Olig2-positive cells; however, they were largely observed in the WMI group than in the sham group. White arrows indicate ferritin^+^CD31^+^ cells in c5 and d5 and ferritin^+^Olig2^+^ cells in f5. Scale bar, 50 *μ*m.

**Figure 6:**
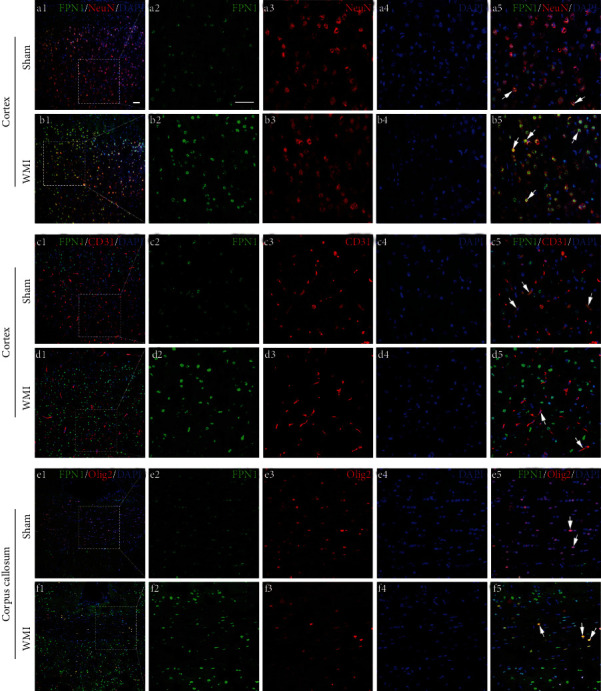
Representative images of immunofluorescence staining for FPN1 (green), NeuN (neuronal marker; red)/CD31 (vascular marker; red)/Olig2 (oligodendrocyte marker; red), and DAPI (nuclear marker; blue) in rat brains on POD 28. FPN1-positive cells were colocalized with NeuN- or CD31- or Olig2-positive cells; however, they were largely observed in the WMI group than in the sham group. White arrows indicate FPN1^+^NeuN^+^ cells in a5 and b5, FPN1^+^CD31^+^ cells in c5 and d5, and FPN1^+^Olig2^+^ cells in e5 and f5. Scale bar, 50 *μ*m.

**Figure 7:**
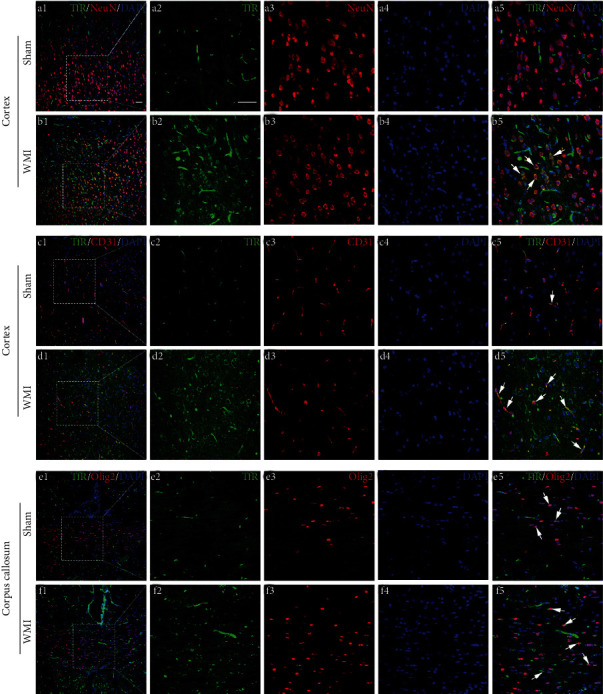
Representative images of immunofluorescence staining for TfR (green), NeuN (neuronal marker; red)/CD31 (vascular marker; red)/Olig2 (oligodendrocyte marker; red), and DAPI (nuclear marker; blue) in rat brains on POD 28. TfR-positive cells were colocalized with NeuN- or CD31- or Olig2-positive cells; however, they were largely observed in the WMI group than in the sham group. White arrows indicate TfR^+^NeuN^+^ cells in b5, TfR^+^CD31^+^ cells in c5 and d5, and TfR^+^Olig2^+^ cells in e5 and f5. Scale bar, 50 *μ*m.

The Western blot results showed that the expression levels of iron-related proteins in the brain were changed after HI. In order to further observe the distribution of these iron-related proteins in the cortex and corpus callosum, we selected the time point with the maximum difference in protein expression (POD 28) for immunofluorescence staining of the brain tissues. The results showed that the ferritin-positive cells were mainly colocalized with the CD31-positive blood vessels in the cortex (Figure 5, c5 and d5) and the Olig2-positive oligodendrocytes in the corpus callosum (Figure 5, f5). In the sham group, there were only rare cells colabeled for ferritin and CD31/Olig2. WMI rats showed an increased number of ferritin^+^CD31^+^DAPI^+^cells or ferritin^+^Olig2^+^DAPI^+^cells in the cortex and corpus callosum (Figure 5). The immunopositive markers of FPN1 were the most granular. They were mainly distributed in the NeuN-positive neurons (Figure 6, a5 and b5) and CD31-positive blood vessels (Figure 6, c5 and d5). The FPN1- and Olig2-positive oligodendrocytes also can be seen in the corpus callosum region (Figure 6, e5 and f5). Moreover, the number of FPN1-positive immunomarkers in the WMI group in the cortex and corpus callosum was significantly higher than that in the sham group. The TfR-positive cells were also colocalized with NeuN-positive neurons (Figure 7, b5), CD31-positive blood vessels (Figure 7, d5) and Olig2-positive oligodendrocytes (Figure 7, e5 and f5) in the cortex and corpus callosum. Besides, TfR-positive immunomarkers in the WMI group in the cortex and corpus callosum were increased compared with those in the sham group.

## 4. Discussion

A change in iron metabolism is one of the pathophysiological mechanisms of hypoxic-ischemic injury in the central nervous system [9]. Although many drugs have been tested in animal models and clinical trials, effective treatments for hypoxic-ischemic WMI are still very limited. Currently, there is some evidence indicating that hypothermia therapy is an effective treatment after HI in premature infants; however, the mortality and disability rates in these patients are still very high [17, 23]. Abnormal iron metabolism occurs in the brain following neonatal HI, and iron-related proteins may play an important role in this process. We adopted a hypoxic-ischemic WMI animal model to explore the expression and localization of iron-related proteins in the brain. Our results showed that the expression of iron-related proteins (ferritin, TfR, and FPN1) changed dynamically after HI in neonatal brains and was mainly expressed in the cortex region in which the distribution of blood vessels was the most obvious. In addition, some proteins were expressed in the cell body and less in the corpus callosum region.

In a previous study of preterm infants, oligodendrocytes (preOLs) dominated the white matter before myelination and were susceptible to HI and oxidative stress [24]. If the preOLs are defective or damaged, remyelination failure and long-term motor, cognitive, and behavioral deficits result [25]. Our study found that the expression of MBP was significantly downregulated after HI, which confirmed the occurrence of WMI.

Iron is a basic requirement for oxidative metabolism, which is crucial for lipid synthesis and normal myelination [26, 27]. Iron homeostasis was found to be upregulated and appeared to regulate myelin metabolism [28]. Our previous research also shows that changes in iron metabolism could affect myelination [14]. But it is still unclear how the abnormal iron metabolism occurred in the hypoxic-ischemic WMI. Cellular iron levels are precisely controlled in physiological conditions. Ferritin, TfR, and FPN1 play an important role in the regulation of iron metabolism. Ferritin is an acute phase reactive protein, which increases in some inflammatory diseases, trauma, and various cardiovascular and cerebrovascular diseases [29]. The increase of ferritin may protect cells against iron-mediated oxidative damage during ischemia-reperfusion [19]. Our results also found that the expression of ferritin was significantly upregulated on PODs 3, 7, 14, and 28, which suggested that iron may accumulate in the brain and affect normal development of the nervous system. FPN1 is the only known iron export protein that could accelerate iron circulation and mobilisation [17]. In this study, we showed that the expression of FPN1 began to increase on POD 7 and was significantly increased on PODs 28 and 56, indicating that iron accumulation may have occurred in the brain area after HI. At this point, FPN1 expression increases compensatory to the iron output to accelerate the iron recycling and reduce iron accumulation, which could also be inferred from the decreased expression of ferritin and TfR on POD 56 compared with POD 28. Moreover, the expression of FPN1 may influence the expression of the other two iron-related proteins. Some studies have found that hypobaric hypoxia decreased hepcidin levels and might cause FPN1 increase, which can lead to iron decrease in liver cells [30]. However, FPN1 levels were actually decreased in the injury model of MCAO [7] and chronic intermittent hypoxia [20]. Therefore, we inferred that the FPN1 expression level depended to some extent on the type of the animal model and whether the pathway was hypoxic or ischemic. Increasing evidence shows that the Tf-TfR pathway may be the main pathway for iron uptake through capillary endothelial lumens and nerve cells [31] and that TfR helps maintain iron metabolism and homeostasis [32]. Our study demonstrated that the TfR expression begins to increase on POD 1 and is significantly increased by PODs 7 and 28. Palmer et al. [33] confirmed that the increased hypoxia-inducible factor 1 expression promotes the expression of TfR1, resulting in an increase in free iron. Therefore, the increase of TfR may somewhat exacerbate the occurrence of iron accumulation. The expression level of ferritin and TfR returned to that of the control group 56 days after HI, although the FPN1 expression was still elevated indicating that there may be other bypass pathways that lead to iron accumulation, and thus, the expression level of FPN1 could not return to that of the control group. The above results showed that ferritin, FPN1, and TfR expressions participate in the regulation of intracellular iron levels after HI, although the changes in iron-related proteins in the brain may be involved in a dynamic process according to the time, nature, and degree of HI; body compensation; and correlation between and influence of different damaged target organs.

Through immunofluorescence staining, we found that iron-related proteins (ferritin, FPN1, and TfR) were mainly distributed in the cortex area and a few in the corpus callosum area, and their expression was more obvious in blood vessels. According to the histochemical study of iron levels in normal and hypoxic-ischemic rat brains by Palmer et al. [33], the earliest areas of hypoxic-ischemic encephalopathy injury were the iron-rich cortical neurons. Furthermore, FPN1 was distributed in the cortex of mice after transient ischemia and in gerbil brains after 5 min of reperfusion for 4 days [34]. Our results are similar to these findings.

Our experiment has some limitations. The specific molecular mechanism behind the changes of these iron-related proteins in the brain requires further in-depth exploration and experimental verification. In addition, other regulatory proteins that maintain iron homeostasis, such as hepcidin, divalent metal ion transporter 1, and ceruloplasmin, may also be involved in the regulation of iron homeostasis, which will be the direction of further studies by our team.

## 5. Conclusions

Our study showed that expression levels of iron-related proteins (ferritin, FPN1, and TfR) in the brain appear to be dynamic after hypoxic-ischemic WMI, which may be inextricably related to the occurrence of iron accumulation. These results provide a new direction for the clinical treatment of WMI.

## Data Availability

The data and analysis in this study could be reasonably acquired from the corresponding authors.
